# *Ebolavirus* Nucleoprotein C-Termini Potently Attract Single Domain Antibodies Enabling Monoclonal Affinity Reagent Sandwich Assay (MARSA) Formulation

**DOI:** 10.1371/journal.pone.0061232

**Published:** 2013-04-05

**Authors:** Laura J. Sherwood, Andrew Hayhurst

**Affiliations:** Department of Virology and Immunology, Texas Biomedical Research Institute, San Antonio, Texas, United States of America; The University of Texas Medical Branch, United States of America

## Abstract

**Background:**

Antigen detection assays can play an important part in environmental surveillance and diagnostics for emerging threats. We are interested in accelerating assay formulation; targeting the agents themselves to bypass requirements for *a priori* genome information or surrogates. Previously, using *in vitro* affinity reagent selection on Marburg virus we rapidly established monoclonal affinity reagent sandwich assay (MARSA) where one recombinant antibody clone was both captor and tracer for polyvalent nucleoprotein (NP). Hypothesizing that the closely related *Ebolavirus* genus may share the same Achilles' heel, we redirected the scheme to see whether similar assays could be delivered and began to explore their mechanism.

**Methods and Findings:**

In parallel we selected panels of llama single domain antibodies (sdAb) from a semi-synthetic library against Zaire, Sudan, Ivory Coast, and Reston Ebola viruses. Each could perform as both captor and tracer in the same antigen sandwich capture assay thereby forming MARSAs. All sdAb were specific for NP and those tested required the C-terminal domain for recognition. Several clones were cross-reactive, indicating epitope conservation across the *Ebolavirus* genus. Analysis of two immune shark sdAb revealed they also targeted the C-terminal domain, and could be similarly employed, yet were less sensitive than a comparable llama sdAb despite stemming from immune selections.

**Conclusions:**

The C-terminal domain of *Ebolavirus* NP is a strong attractant for antibodies and enables sensitive sandwich immunoassays to be rapidly generated using a single antibody clone. The polyvalent nature of nucleocapsid borne NP and display of the C-terminal region likely serves as a bountiful affinity sink during selections, and a highly avid target for subsequent immunoassay capture. Combined with the high degree of amino acid conservation through 37 years and across wide geographies, this domain makes an ideal handle for monoclonal affinity reagent driven antigen sandwich assays for the *Ebolavirus* genus.

## Introduction

Viruses are usually experts in energetic economy, employing multiple copies of a relatively small repertoire of key architectural templates to assemble infectious particles comprising outer coat/envelope superstructures and internal substructures such as matrix and nucleocapsid [1], [2]. Consequently, to formulate a virus specific sandwich assay only a single antibody clone specific to a surface exposed, preferably highly conserved epitope on one of these polyvalent virus components should be required. Such a system can be thought of as a monoclonal affinity-reagent sandwich assay (MARSA) which is an evolution of the term single antibody sandwich ELISA (SAS-ELISA) meant to encompass other forms of antigen recognition elements [3] non-ELISA based methods of signaling [4], and to avoid confusion with single molecule analyses. MARSAs have already been used to detect several disease relevant oligomers, though sparingly for emerging viral pathogens; the scrapie prion protein isoform PrP^Sc^ [5], Alzheimer disease amyloid-β protein [6], Parkinson's disease α-synuclein protein [4], [7], *Plasmodium malariae* sporozoites [8] and hemagglutinin content in influenza vaccines [9]. The polyvalent display of the antigenic epitope on large protein oligomers should ensure a very strong interaction with surface immobilized captor antibody increasing the sensitivity for even mediocre affinity antibodies through a Velcro like effect *via* massively parallel avidity. This condition is met provided the assay conditions preserve antigen polyvalency which is especially important for enveloped viruses since detergents used to reduce non-specific binding or to inactivate the agent, may destroy the envelope. Such destruction may perturb glycoprotein (GP) presentation and fragment the underlying matrix to require two different non-competitive antibodies for antigen detection as exemplified by Ebola VP40 and GP [10], [11]. As a model system for exploring what combinations of antibodies and emerging viral antigens might yield functional MARSA systems, we have been targeting Filovirus preparations at biosafety level 4 (BSL-4) with our semi-synthetic llama single domain antibody (sdAb) single pot library.

The Filoviruses or family *Filoviridae* primarily contain a *Marburgvirus* species, four African *Ebolavirus* species; *TaÏ Forest ebolavirus*, *Sudan ebolavirus*, *Zaire ebolavirus* and *Bundibugyo ebolavirus* (herein referred to as Ivory Coast, Sudan, Zaire and Bundibugyo viruses) plus *Reston ebolavirus* (herein Reston virus) from the Philippines [12]. Filoviruses are human health threats *via* natural outbreaks in equatorial Africa [13], [14], [15] and also pose a threat to other continents *via* importation by infected tourists as recently occurred in Europe [16] and the United States [17]. Marburg virus was actually first discovered in Europe following importation of experimental non-human primates from Uganda [18] and Reston virus (not pathogenic for humans to date) has arrived in Europe and the U.S multiple times from imported Philippine non-human primates. Filoviruses are also potential biological weapons [19], now categorized as Tier 1 threats within the Category A list of select agents [20], and though they are not particularly contagious under normal circumstances, an outbreak in the U.S. is highly likely to induce fear, major societal and economic upheaval owing to public misconceptions of the threat [21], [22]. Capable of causing hemorrhagic fevers and with case fatality rates of >90% depending on strain and outbreak circumstances, the Filoviruses are zoonoses. Spillover results from human contact with infected bushmeat [23] or specific species of bats that may act as reservoir hosts [24], [25], [26] and can then be transmitted person to person *via* close contact with infectious bodily fluids. Serum surveys of orangutans in Indonesia [27], bats in Ghana [28] and China [29], plus sequence based interrogation of tissues from a bat die off in Spain [30] indicate that Filoviruses or Filovirus-like agents maybe more widely distributed than originally thought making these very relevant model targets for our studies.

Though recent advances in the generic ruggedization of multi-domain IgG antibody formats is occurring [31] especially in systems compatible with display and selection [32], [33], sdAb remain the only antibody format that are almost always rugged and refoldable without further engineering [34], [35]. Such properties can enable highly regenerable immunoassays [36], and may confer unlimited shelf-life potential to diagnostics in cold-chain free environments. SdAb are derived by cloning the variable domains of heavy chain only antibodies of Camelids and cartilaginous fish (for reviews see [37], [38]). Their small size and often long complementarity determining region (CDR) 3 loops can be of benefit in targeting cryptic [39] and concave epitopes [40], and they have already shown great promise in several antiviral studies as monomers, fusions, and multimeric formats [41]. Since sdAb are so modular and inexpensive to produce from *E. coli* in bountiful amounts we still favor these versatile little antibodies over other formats for their simplicity, ease of use and cost effectiveness.

Single-pot libraries enable a rapid response to any antigen, bypassing time-consuming immunizations, concerns of agent lethality plus agent inactivation and validation that would be required before administration to animals outside of containment. Importantly, by panning on live agent rather than recombinant components, one is almost guaranteed to isolate binders that recognize authentic agent since epitopes will be in native conformations and protein surfaces presented in natural juxtapositions. Single-pot libraries can yield antibodies specific for epitopes that may not necessarily be targeted by a natural immune response [42] and can yield hundreds of diverse clones against individual small protein antigens [43], [44], [45]. However, most laboratories' single-pot libraries may not necessarily be endowed with the diversity and functionality of the best repertoires [46] resulting in a decreased spectrum of antigen binding clones often with mediocre affinities as opposed to using an immune approach. Indeed, though our own single-pot sdAb library was fairly consistent in yielding a handful of binders to various protein targets and even facilitated co-crystallization of a previously uncrystallizable antigen [47], affinities for the sensitive detection of proteins *via* antigen capture assays using non-competitive pairs of antibodies were somewhat lacking [48]. It was therefore a surprise when we succeeded in selecting four unique sdAb against live *Marburgvirus Musoke* that could each be used as both captor and tracer to formulate very sensitive MARSA systems [49]. The assays were able to recognize all three of the *Marburgvirus* strains to hand; *Musoke*, *Ravn* and *Angola*, were specific for nucleoprotein (NP) and were enhanced 100 fold by exposure to detergent known to destroy the virus particles yet leave polymeric nucleocapsid filaments intact [18].

Based on our serendipitous success with *Marburgvirus* sdAb and aware that the closely related *Ebolavirus* genus share enough morphological [50], and compositional similarities [51], [52], [53], we hypothesized that selections of sdAb from the same single-pot library using similar methods should also yield useful MARSA systems based on *Ebolavirus* NP recognition. Recently using hapten mediated display and pairing a single sdAb was selected on *Ebolavirus* Zaire from a retrofitted version of this library [54] and the present study is a more thorough evaluation of the response to four *Ebolavirus* species using conventional display [55]. We selected 17 unique sdAb on four *Ebolavirus* species that could all formulate sensitive MARSAs and characterized their cross-reactivities on virus and recombinant NP to help predict likely future strain specificity. We define the major domain recognized by our sdAb and show that two immune shark sdAb [56] also converge on this region and can formulate MARSAs though both appeared less sensitive than a comparable llama sdAb. We show loss of recognition of NP derived from gamma irradiated/beta-propiolactone inactivated virus indicating sdAb can preferentially identify disease relevant antigenic forms of NP. We rationalize why the NP C-terminal region is compatible with MARSA and discuss its potential for evolution to suggest it makes a potent Achilles' heel for Filovirus identification.

## Results and Discussion

### Each *Ebolavirus* species selects a different panel of sdAb from the single-pot semi-synthetic llama library

**Figure 1:**
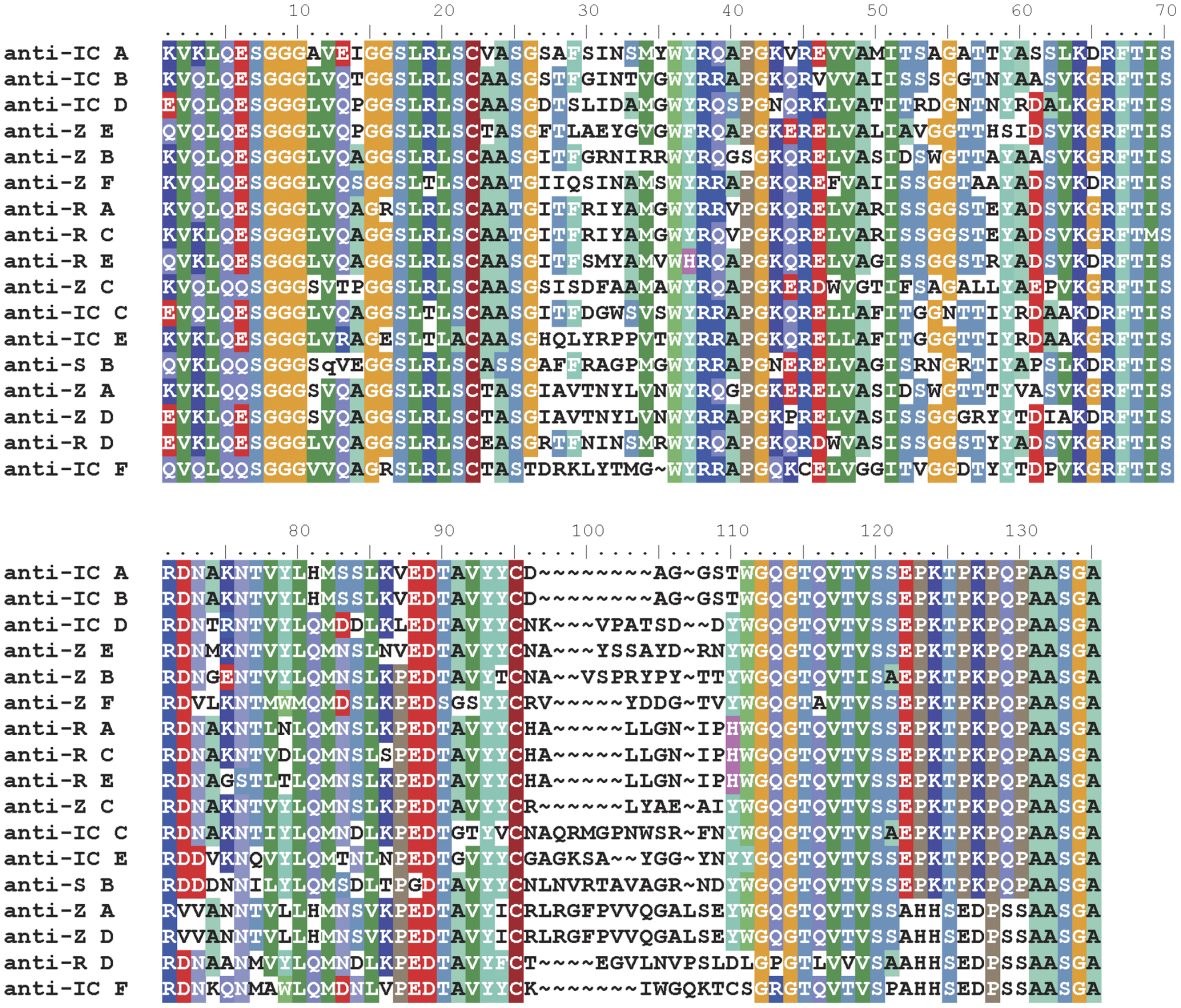
Predicted amino acid sequences of sdAb selected on four *Ebolavirus* species. Unique sdAb genes selected on Ivory coast (IC), Reston (R), Sudan (S) or Zaire (Z) viruses were sequenced and translated using BioEdit [116] and the resulting proteins aligned using MultAlin [117].

Figure 1 shows the predicted amino acid sequences of unique sdAb that were generated by phage panning on live virus preparations of four of the five species of *Ebolavirus* and Table S1 shows their frequency of occurrence. Sudan virus appeared to select only 1 clone that possessed an amber codon at amino acid position 12, which we mutated to bypass the need for supE mediated suppression to enable high level expression for sdAb characterization. The three other viruses all selected multiple clones, although 3 of the 4 anti-Reston sdAb (A, C and E) are very similar in CDR composition. Between panels, some sdAb shared CDRs e.g. anti-Sudan sdAb B shares CDR1 with anti-Zaire sdAb G, and this may be a reflection of the CDR shuffling method we used to make the original library [48] where a relatively small 1e+6 repertoire was hypermutated by error prone PCR and assembly of CDRs 1+2+3, 1−2+3, 1+2−3 and 1−2−3 (where + indicates fragment and–indicates contiguous sequence).

### Each sdAb is able to act as both captor and tracer in antigen capture assays

**Figure 2:**
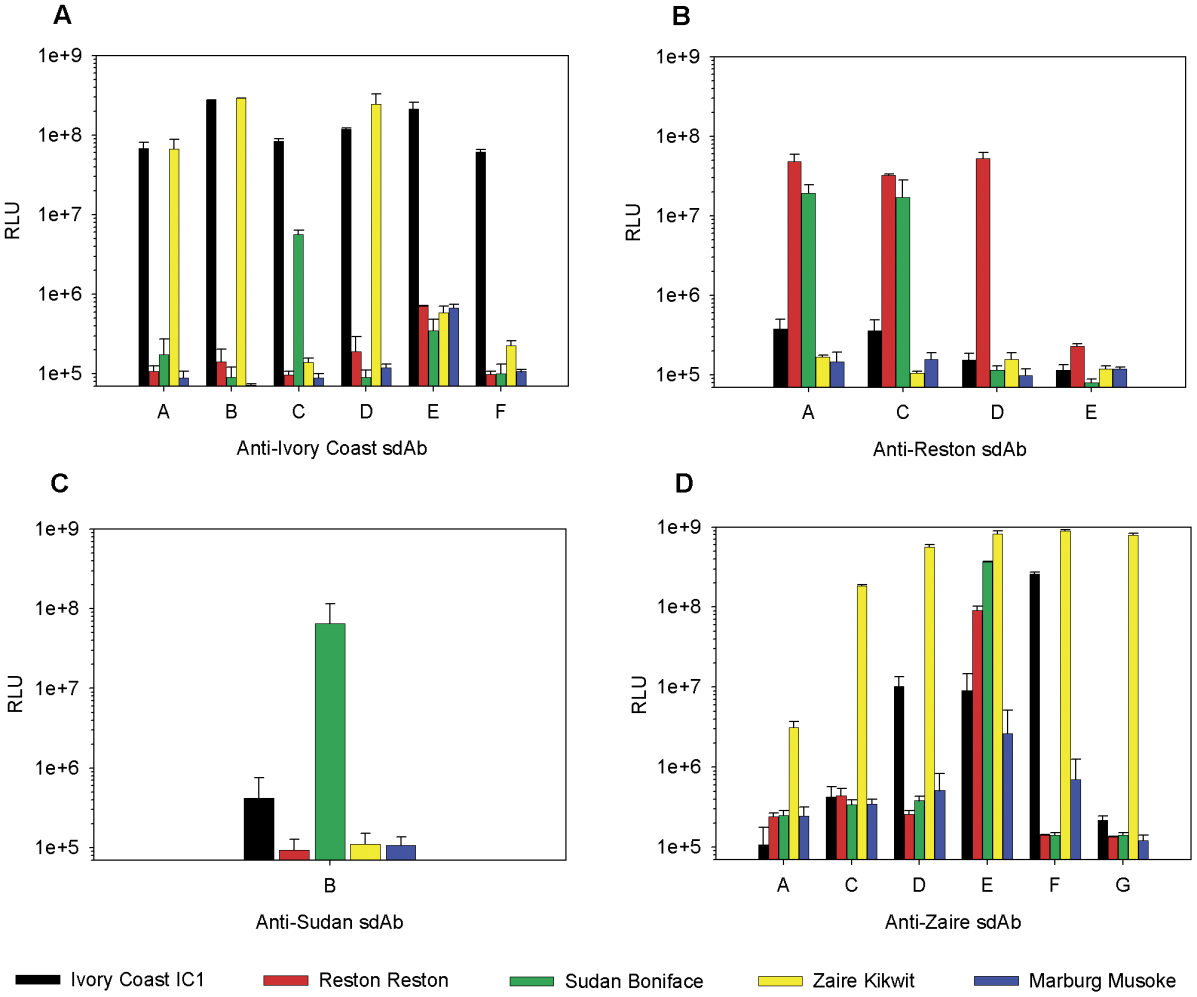
Specificities of monoclonal affinity reagent sandwich assays (MARSA) employing sdAb as captors and phage displayed sdAb as tracers. Individual sdAb selected on (A) Ivory Coast, (B) Reston, (C) Sudan or (D) Zaire viruses were passively immobilized to ELISA wells to serve as captors for 1e+4 pfu of Ivory Coast (black), Reston (red), Sudan (green), Zaire (yellow) or Marburg (blue) virus preparations incubated in 0.1% Triton X-100. Detection used the same sdAb clone as a phage displayed tracer, followed by anti-phage-HRP conjugate and chemiluminescent substrate. The experiment was performed once and the error bars represent the maximum and minimum between duplicate ELISA wells.

We began assessing the mechanism of antigen recognition by examining if a single antibody clone is able to detect target as both captor and tracer, ie. in the same manner as our Marburg virus MARSA, as opposed to needing two different sdAb clones binding non-competitively. Purified anti-*Ebolavirus* sdAb proteins were employed as captors, with phage displayed sdAb plus anti-phage M13-HRP conjugate as tracers, and the combinations used to detect a constant amount of virus (1e+4 pfu) exposed to 0.1% Triton X-100. Each clone could detect that species upon which it was selected, with several sdAb exhibiting varying degrees of cross-reactivities with one or more species of *Ebolavirus* indicating conserved epitope(s) were being targeted (Fig. 2). That we did not isolate the same cross-reactive clones from selections on different viruses may indicate that cognate virus selects for the optimal clones during panning, though will tolerate sufficient binding by other clones during screening.

**Figure 3:**
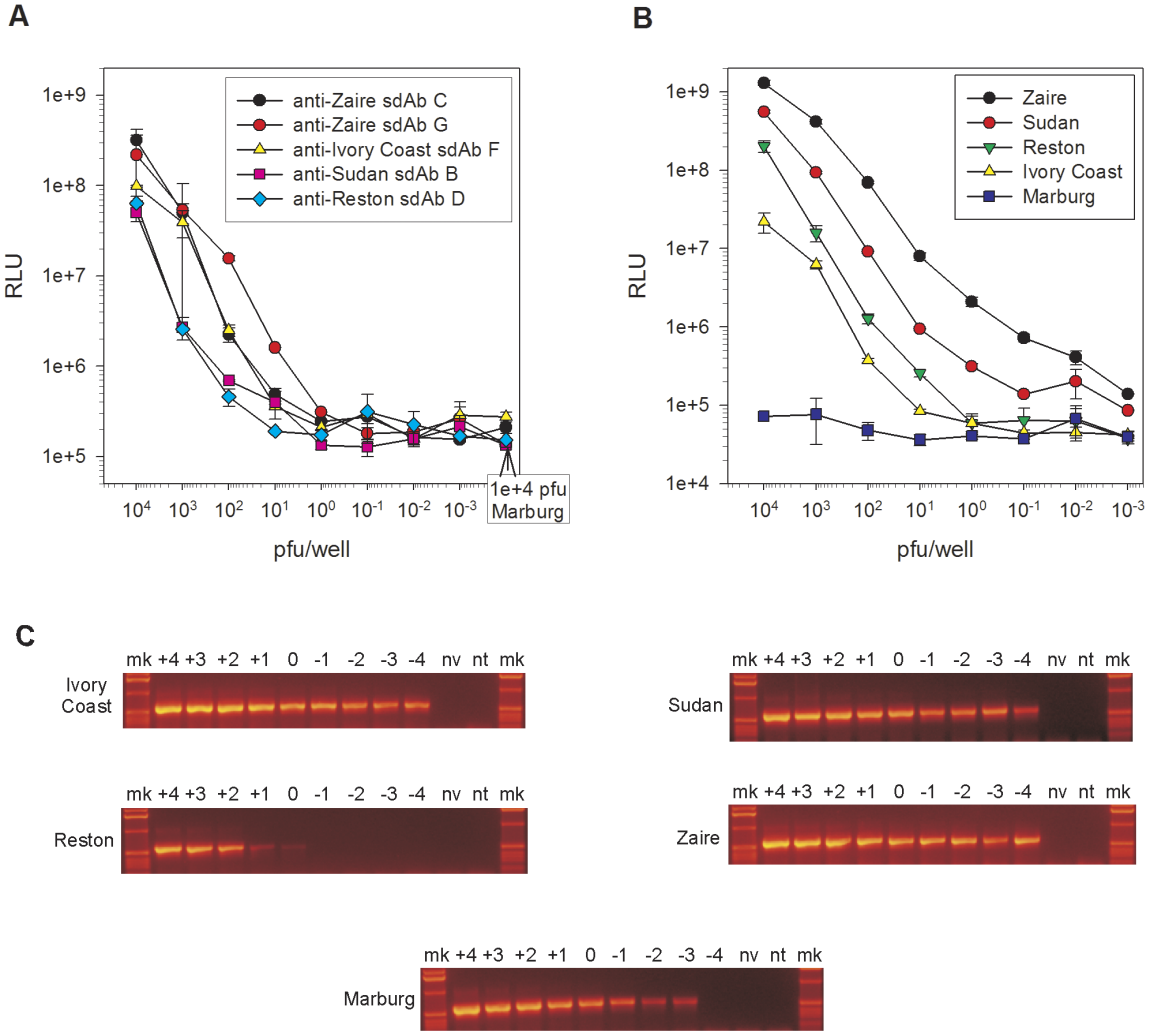
Limits of detection of specific and cross-reactive anti-Ebolavirus sdAb. A) What appeared to be the most specific sdAb for each virus were used as captor and phage displayed tracer to titrate cognate virus from 1e+4 to 1e-3 pfu/well. Background signal was established with a set 1e+4 pfu/well of *Marburgvirus* Musoke. Duplicate wells were utilized for each virus dilution and error bars represent the maximum and minimum values. B) The anti-Zaire sdAb E which appeared to be the most cross-reactive clone was used to titrate all of the viruses including negative control Marburg from 1e+4 to 1e-3 pfu/well. Duplicate wells were utilized for each virus dilution with error bars representing maximum and minimum values. C) PCR titration of Trizol extracted dilutions of virus using universal Filovirus primers [57] to yield a 594 bp band with nv being no virus control and nt being no template control. Amount of virus subjected to RT-PCR (e pfu) across top of gel, mk being marker lanes.

We titrated what appeared to be the most virus specific clones and the most cross-reactive clone for their lower limits of detection to reveal high sensitivities despite the lack of assay optimization. Signal to noise ratios of 10 could be obtained at 10 pfu for anti-Zaire sdAb G, 100 pfu for anti-Zaire sdAb C and anti-Ivory Coast sdAb F, while a 1000 pfu were required for anti-Sudan sdAb B and anti-Reston sdAb D to generate signal (Fig 3a). When titrating the cross-reactive anti-Zaire sdAb E on cognate virus we failed to titrate out at 1e-3 pfu, though non-cognate species required much larger amounts to give signal to noise ratios of 10, with 10, 100 and 500 pfu per well required for Sudan, Reston and Ivory Coast viruses respectively (Fig 3b). RT-PCR detection using the Filoviral universal primers specific for NP followed by agarose gel analysis [57] was used to benchmark the immunoassay (Fig. 3c). Though anti-Zaire E sdAb showed comparable sensitivities on Zaire virus, antibody engineering will be required to improve sensitivity for the other Ebola species before the assay is ready for validation on a broad set of clinical samples.

### Nucleoprotein appears to be the major antigenic determinant for all of the sdAb

**Figure 4:**
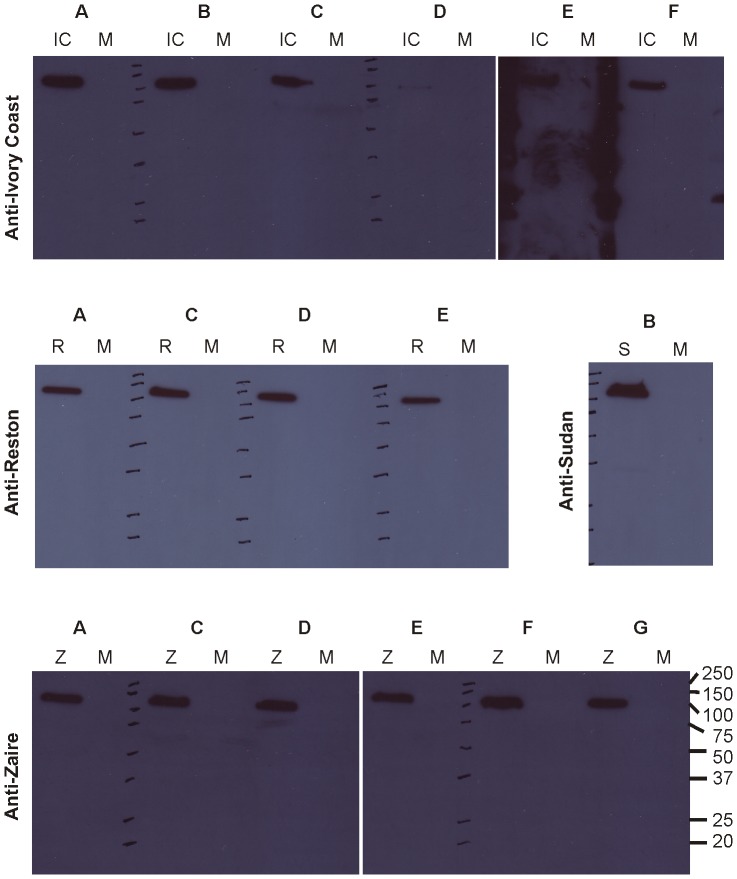
Probing viral components with sdAb-alkaline phosphatase fusion proteins to identify the antigenic targets. Each set of sdAb-AP fusions was used to probe western blot membranes of cognate (IC = Ivory Coast, R = Reston, S = Sudan, Z = Zaire) or control (M = Marburg) virus preparations to indicate all sdAb appeared to recognize an approx. 100 kDa band likely to be nucleoprotein.

We first repeated antigen captures assays using a fixed concentration (100 nM) purified sdAb-AP (alkaline phosphatase) fusion proteins as tracers in place of phage to show the same trends in cross-reactivity and relative sensitivities overall (Fig. S1). Western blots of infected cell lysates capturing the complement of Filoviral proteins were then probed with the sdAb-AP fusions to indicate a band migrating at 100 kDa, was the predominant antigen recognized (Fig. 4). Though NP is calculated to be an approximately 85 kDa antigen, it migrates anomalously in SDS-PAGE gels owing to two highly acidic regions [58]. That the sdAb were capable of binding antigen that had been boiled, reduced and electrophoresed through an SDS gel indicated the epitopes were likely, though not necessarily guaranteed [59], to be linear and non-conformational.

To confirm that the primary targets were indeed NP, the relevant genes were cloned (Sudan) or assembled synthetically (Ivory Coast). NP of Bundibugyo, a virus we did not have access to, was also assembled synthetically to gauge if our binders were likely to be cross-reactive to it. The genes were initially expressed with C-terminal His tags in *E. coli* along with our pre-existing NP clones for Zaire virus and negative control Marburg [49]. Crude lysates were first western blotted with anti-His to confirm all of the proteins were expressed and then a series of new blots were probed with each of the sdAb-AP fusions (Fig. S2). All of the NP proteins are well expressed except for Reston which is not prominent by Coomassie staining and weak with anti-His probing, so we must be careful not to over-interpret relative cross-reactivities based on antigens that are not normalized for concentration. Each of the sdAb-AP fusions react with cognate NP proving that NP is the major target for these sdAb. Since *E. coli* normally lacks the machinery to glycosylate proteins, this also shows that glycosylation of mammalian cell expressed NP [60], [61] is not required for binding.

All clones demonstrate cross-reactivities that, for the most part, track well with the sandwich ELISA data. Anti-Ivory Coast sdAb A, B, C, D demonstrate cross-reactivity to Bundibugyo NP, predicted to be the closest relative by phylogenetic analysis [62]. Binding of anti-Ivory Coast sdAb E to a plethora of *E. coli* proteins in addition to NP indicate this is a sticky or non-specific clone and we did not study this sdAb further. Anti-Sudan sdAb B retains specificity against just Sudan while the anti-Reston sdAb all cross-react between Ivory Coast, Zaire (though sdAb A and C are weak) and Sudan (with the exception of sdAb D). The anti-Zaire sdAb E is the only clone showing strong cross-reactivity to all species except *Marburgvirus* though anti-Zaire sdAb F is fairly cross-reactive too. All anti-Zaire sdAb except C exhibit cross-reactivity with Ivory Coast, and all except sdAb A react with Bundibugyo. Overall therefore, *Ebolavirus* NP comprises a mix of specific and cross-reactive epitopes as seen for a human Fab derived from a survivor of the Zaire Kikwit 1995 outbreak from repertoire cloning and phage display selection on gamma-irradiated virus [63], and mouse monoclonal antibodies generated *via* hybridomas from mice immunized with recombinant NP [64], [65], [66].

### Rationalizing the single antigenic specificity of the selected sdAb

*Ebolavirus* particles are relatively large, pleiomorphic enveloped filaments of almost 1000×100 nm [50], [67] often exhibiting polyploidy [68]. Particles mainly consist of 7 virus encoded proteins in varying amounts estimated by analysis on *Marburgvirus* [53] and *Ebolavirus* [52] to be VP40 (4594/3600 copies), VP35 (2140/2686), VP24 (1400/1208), NP (1062/625), VP30 (837/833), L (56/47) and GP (54/144). There are also very small numbers of specific host proteins present [69] and sGP has potential to be a minor structural component [70]. GP is displayed on the lipid envelope which encapsulates the VP40 matrix which surrounds the ribonucleoprotein complex bearing NP, the other viral proteins and 19 kb single stranded negative sense RNA genome [67], [68]. With such a large array of proteins in live virus particles why are our sdAb specific to a single internal antigen that is not even the most frequent component? Direct adsorption of proteins to polystyrene surfaces used for panning can cause large amounts of protein denaturation [71] and adsorption of virus has been shown to release the contents of influenza A [72] and we would not be surprised if a portion of Ebola virus particles underwent virolysis, thereby exposing internal antigens. Since the washing procedures also used 0.1% Tween-20 we may also have further disrupted the particles during panning and perhaps stripped away loosely attached nucleocapsid proteins leaving a string of NP attached to the genome. Solution phase panning on intact and differentially disrupted virions might address these perplexing questions and allow us to isolate sdAb to the full complement of other antigenic landscapes that make up the virus particles and generate a range of MARSAs with unique antigen specificities.

### The C-terminus of nucleoprotein is the primary determinant for sdAb recognition

**Figure 5:**
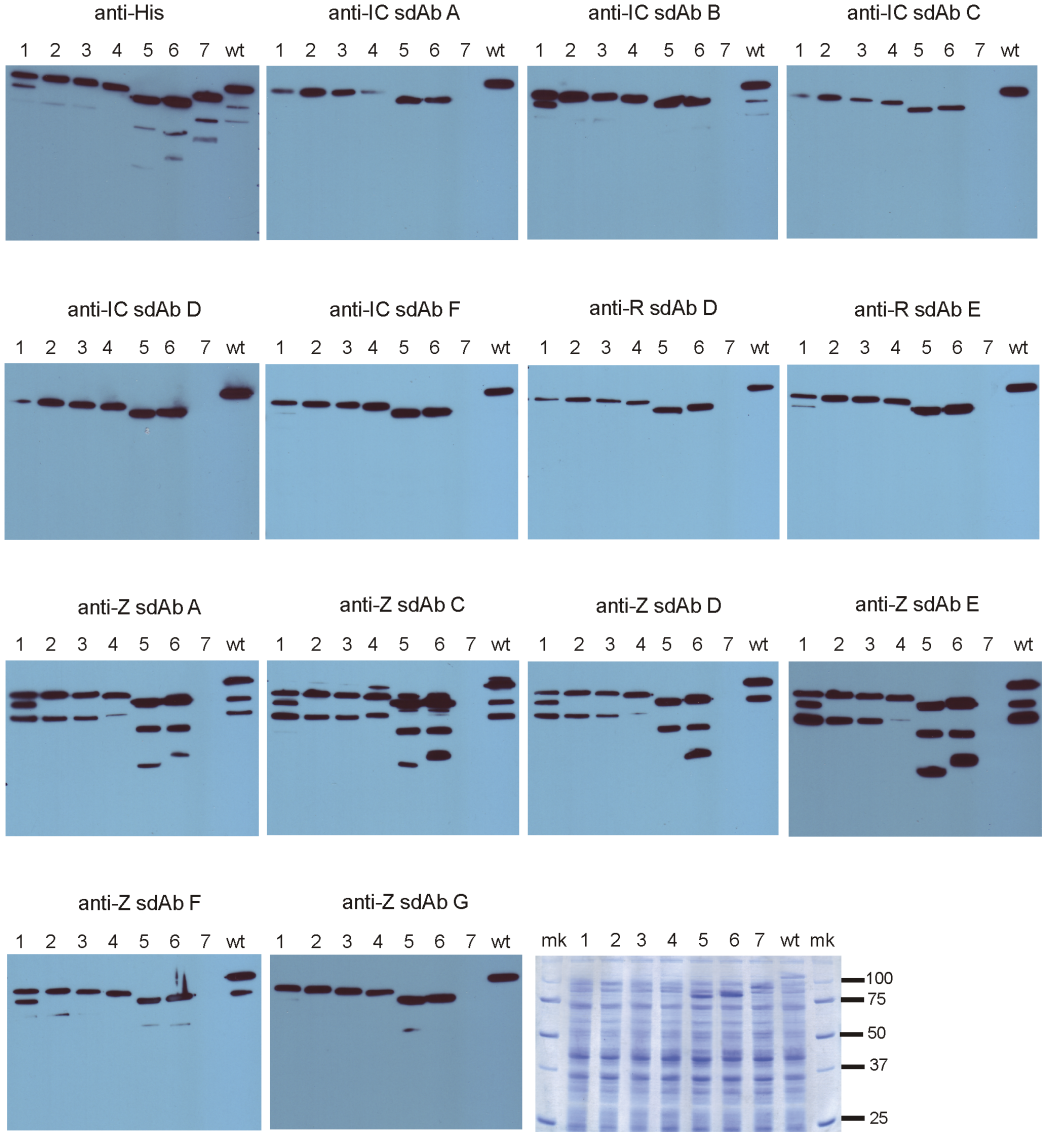
The C-terminus of NP is the major antigenic determinant for Zaire virus reactive sdAb. Deletions of 105 amino acid residues spanning the entire Zaire NP gene N- to C-terminus (1–7) and a full-length gene (wt) were expressed in *E. coli* and probed with anti-His-HRP to confirm expression and then with each of the Zaire reactive/cross-reactive sdAb-AP fusions; IC = Ivory Coast, R = Reston, Z = Zaire. A Coomassie stained gel served to indicate an equivalent amount of lysate was loaded per lane. Deletion 7 is that of the C-terminal region.

In order to begin elucidating where the sdAb bind NP we made deletions of approximately 105 amino acid residues along the length of the recombinant Zaire NP and probed the resulting crude *E. coli* expression lysates with those clones most reactive to the Zaire NP (Fig. 5). Smaller molecular weight products might reflect proteolytic degradation of recombinant protein or internally initiated translation products that retain the relevant epitope(s). All of the clones tested lose reactivity completely when the C-terminal 105 residues are absent. Commenting on panels of antibodies generated by mouse immunizations with recombinant NP, Saijo and colleagues conclude that both *Ebolavirus* and *Marburgvirus* antibodies are only useful in capture assays if they bind the C-terminal 100 amino acids [73]. In these works, the authors employed different captor and tracer antibodies, usually mouse monoclonals as captors and rabbit polyclonals as tracers, for both *Ebolavirus* [64], [65], [66] and *Marburgvirus* detection [73] in contrast to our single affinity reagent driven system. The recent high resolution structures of Filovirus particles indicate that NP exposes its C-terminus at regular intervals along the length of the nucleocapsid in both viruses [67], [74]. Such a regularly repeating array of several hundred NP molecules would be likely to afford a highly avid molecular Velcro like antigen when binding capture antibodies, explaining why the assays are so sensitive without immunization or optimization.

### Generating a BSL2 screen for predicting antigen recognition

While we had previously found *E. coli* derived NP lacking tags [75] to be suitable as a crude polyvalent surrogate for capture of Zaire virus and *Marburgvirus Musoke* [54], we decided to generate small-scale mammalian equivalents of these and other species as more faithful representations that are amenable to high quality imaging [76], [77] and facile purification [78] for future structural studies. Such recombinant material would be very useful in predicting the likely cross-reactivities of antibodies even if viral strains are not made available or cannot be grown *in vitro*, as long as genetic information is available. As noted by others in developing nucleic acid based tests for Filoviruses, it is important that public databases of genetic information are kept up to date to ensure developed assays recognize contemporary strains [79], [80].

Human codon optimized NP genes were transiently expressed in human embryonic kidney cells (HEK 293T) and lysates analyzed for the production of Coomassie stained bands of the expected molecular weight (Fig. S3a) that correlated with immune-reactivity when probed with our cross-reactive anti-Zaire sdAb E as an AP fusion (Fig. S3b). A constant amount of the lysates were employed in the phage based capture assay of the clones to reveal that the cross-reactivity and specificity trends were essentially conserved (Fig S3c-f) when compared with the live virus based experiment performed previously (cf. Fig. 2). The data indicate that several sdAb are likely to bind Bundibugyo virus NP in this sandwich assay format, particularly the Ivory Coast and several Zaire clones including the pan-active anti Zaire sdAb E, also confirming the western blotting of *E. coli* extracts performed previously (cf. Fig. S2). We exercise caution in not trying to estimate likely sensitivities based upon this material as we did not purify or quantify the recombinant protein and only suggest using this as a rough guide to predict potential specificities.

### Comparison of non-immune llama sdAb with shark immune sdAb

**Figure 6:**
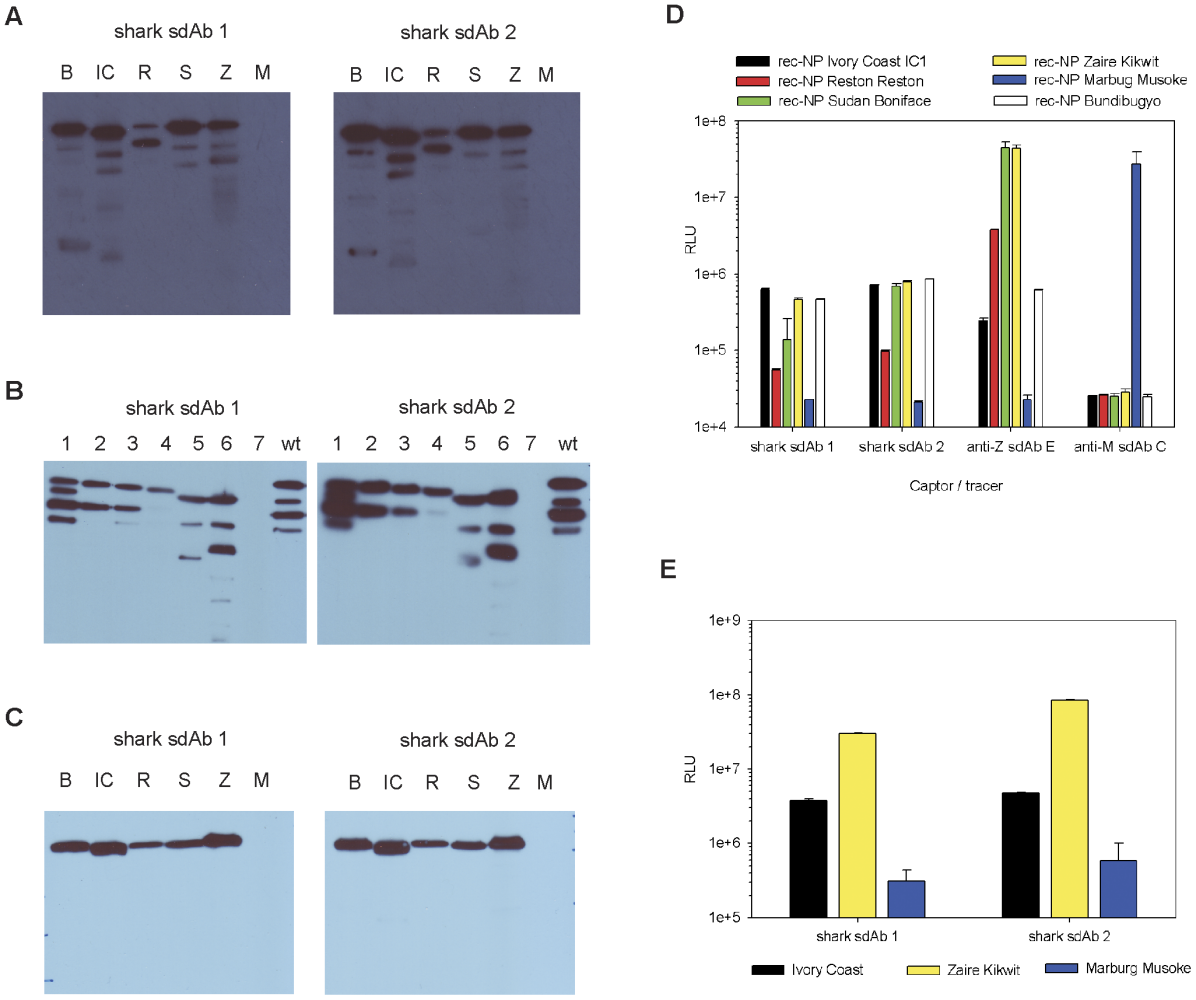
Shark sdAb also bind the NP C-terminal region and are able to form MARSAs. A) Confirming shark sdAb bound the *E. coli* rec-NP preparations as sdAb-AP fusions [Bundibugyo (B), Ivory Coast (IC), Reston (R), Sudan (S), Zaire (R), and negative control Marburg (M)] before, B) identifying the C-terminal domain was the major antigenic determinant by probing on the *E. coli* expressed Zaire rec-NP deletion mutants (notation of deletions as for figure 5, with #7 the C-terminal deletion mutant). C) Confirming shark sdAb bound our recombinant HEK 293T preparations as sdAb-AP fusions by western blot before, D) employing them both in the AP based MARSA to reveal that shark sdAb can indeed form a monoclonal capture ELISA, though they appear less sensitive than the positive control llama anti-Zaire E sdAb for Zaire, Reston and Sudan rec-NP preparations. E) Employing 1e+4 pfu/well of virus in the sandwich assay to reveal the shark clones can bind Ivory Coast in a MARSA format.

Goodchild and colleagues had previously generated two closely related IgNAR derived sdAb specific for *Ebolavirus* NP [56] (DSTL096 and DSTL097, herein referred to as shark sdAb 1 and shark sdAb 2) and we wished to benchmark our clones against these to gauge how worse our single-pot clones were to immune clones. The authors had immunized two nurse-sharks three times over the course of several weeks with 0.25 mg amounts of gamma irradiated sucrose gradient pure *Zaire ebolavirus* to illicit seroconversion. Resulting immune phage display libraries were subtractively panned on inactivated virus preparations captured with pre-existing mammalian polyclonal anti-Zaire IgG to yield one unique clone from each shark that differed by 7 amino acids, 6 in CDR 3. Shown to bind Zaire NP by western blot the authors employed them either as captors or tracers with rabbit polyclonal immunoglobulin as corresponding tracers and captors for virus detection trials to reveal recognition of all species save Ivory Coast. To establish a MARSA for comparison we first assembled synthetic shark sdAb genes and produced them both as sdAb, sdAb-AP and phage displayed sdAb. Employing the sdAb-AP to probe the crude *E. coli* expressed NP preparations revealed both shark clones react with all of the *Ebolavirus* species including Ivory Coast (Fig. 6a). Probing the NP deletion mutants also revealed both shark sdAb lose binding activity in the absence of the C-terminus (Fig. 6b) indicating that they would each likely be useable as both captor and tracer in the same assay. To show this, we first confirmed the shark sdAb were capable of recognizing our HEK293T recombinant NP by western blot (Fig. 6c) before using them in a capture format (Fig. 6d) to show specific capture of all *Ebolavirus* recombinant NP, alongside control llama sdAb anti-Zaire E. Although these are by no means particularly quantitative assays we demonstrate the shark clones bind Ivory Coast material and would be likely to bind Ivory Coast virus especially given the 100 fold higher reactivities to the closely related Bundibugyo virus over cognate Zaire virus shown previously (Fig. 5 of [56]). We confirmed this with demonstration of live Ivory Coast virus binding relative to cognate Zaire virus binding with a Marburg virus negative control, all at 1e+4 pfu per well (Fig. 6e). We wonder if the mammalian IgG the authors used in their assay has somehow discriminated between species and introduced an artifact into the analysis of sdAb specificity. Differences in findings could also be due to titration of virus stocks used between labs which is known to be particularly fickle for Filoviruses [81] and variation in the ratio of virions to virus particles [82] also likely to vary with local propagation conditions. These factors should stimulate a global push to assemble a central standard collection of live Filovirus samples and reagents to benchmark assays between BSL-4 laboratories, and points to another advantage of sdAb in that their recombinant nature means the gene sequence is immortalized *in silico* for all to use.

**Figure 7:**
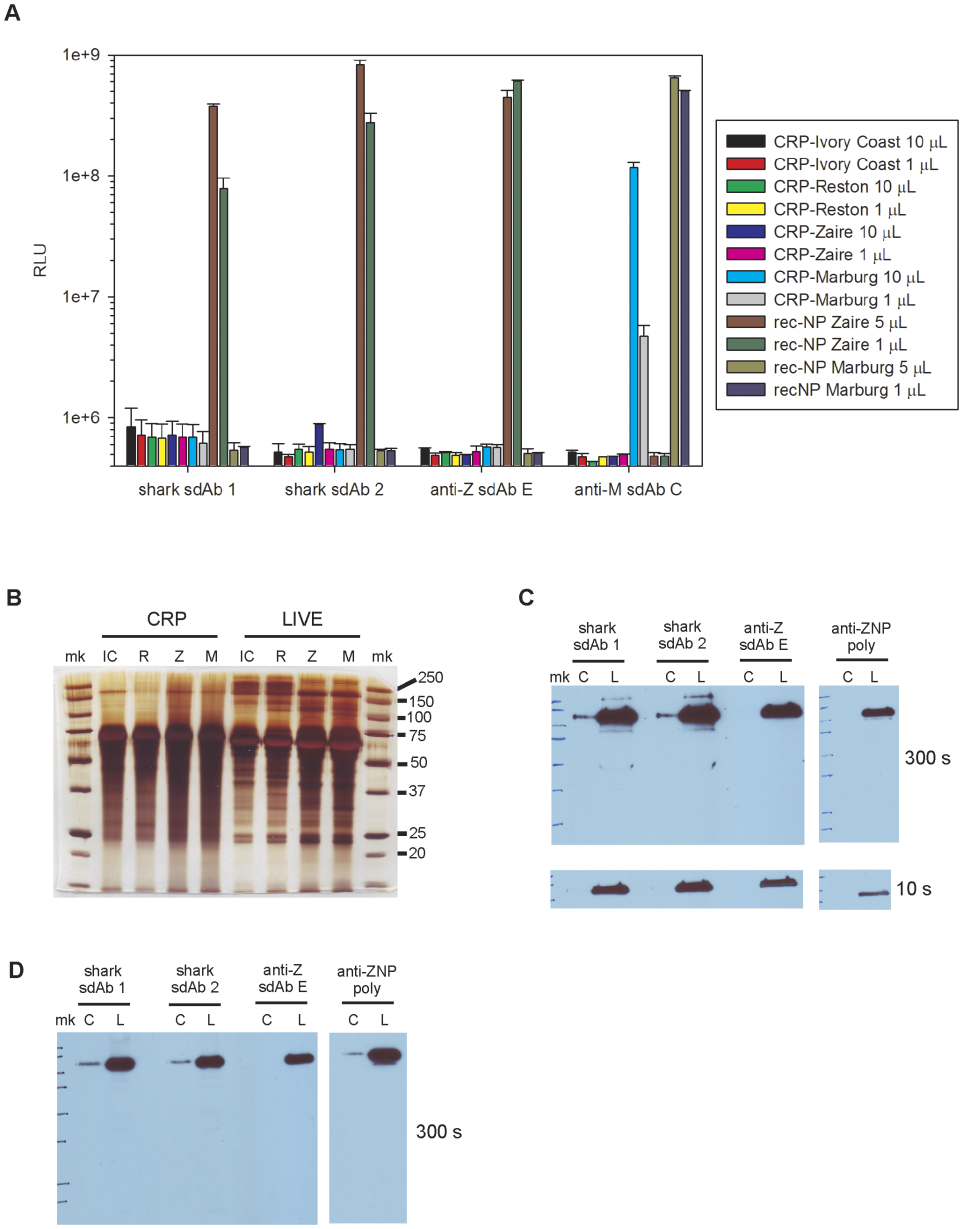
NP epitope loss within inactivated virus standards. A) Failure of Critical Reagents Program (CRP) gamma irradiated/beta-propiolactone inactivated *Ebolavirus* preparations to signal in the phage based MARSA when compared with HEK 293T recombinant NP lysates (rec-NP). Pfu per well in 10 µL samples- Ivory Coast, 4.1e+3; Reston, 1.1e+5; Zaire, 1.7e+5; Marburg Musoke 3.6e+4. The error bars represent the maximum and minimum values of duplicate ELISA wells. B) Silver stained SDS-PAGE of inactivated (CRP) and virus infected Vero supernatants (LIVE) loaded at equivalent volumes to examine protein content; Ivory Coast (IC), Reston (R), Zaire (Z), Marburg (M), markers (mk) indicated in kDa. C) Western blots of equivalent titers of inactivated Zaire CRP material (C: 4.3e+4 pfu) versus live supernatants (L: 2.5e+4 pfu) to examine NP antigenicity by probing with the two shark sdAb, anti-Zaire sdAb E or anti-Zaire NP specific polyclonal rabbit serum (anti-ZNP poly). D) Western blotting of equivalent titers of inactivated Zaire CRP material (C: 4.3e+4 pfu) versus live freeze-thaw lysates (L: 2.3e+4 pfu) probed as for (C).

On that note, we attempted to use virus preparations from the Critical Reagents Program (CRP) that were gamma-irradiated and beta-propioloactone (BPL) inactivated supernatants, as standards to further compare shark and llama sdAb. The anti-Marburg sdAb C detected CRP Marburg material by MARSA while llama anti-Zaire E and the two shark sdAb did not appreciably detect CRP Ebola material (Fig. 7a). That the CRP Zaire virus is present at 17 fold higher concentrations as used in our previous cross-reactivity assays yet fails to signal indicates either a loss of NP polyvalency and/or antigenicity. Silver staining of equivalent volumes of CRP material versus our own live virus supernatants (as used in Figs. 2 and 3) revealed cell culture proteins to be present throughout, though somewhat smeary in the CRP preparations (Fig. 7b). Western blotting of equivalent titers of CRP Zaire material versus our own supernatant revealed that the shark sdAb and a rabbit polyclonal specific for Zaire NP could only detect NP in CRP material after over exposure (300 s) yet were able to detect our live material after brief exposure (10 s) (Fig. 7c). However, the llama anti-Zaire sdAb E was only faithful to live material and did not detect CRP NP antigen, reflecting the advantage of live virus selections in ensuring stringent recognition of authentic antigen. Repeating the western blotting using our freeze thaw lysates (as used in Fig. 4) loaded at equivalent titers again showed the same trend, with recognition of CRP material requiring overexposure of the blot to show weak binding by shark sdAb 1 and 2 but not the anti-Zaire sdAb E (Fig. 7d).

While gamma irradiation *can* conserve viral antigenic epitopes [83], and irradiated Filoviruses [84], [85] have been used to detect polyclonal antibody responses in animal challenges and human survivors [86], recent studies reveal the doses used for Filovirus inactivation (approx. 3e+6 rads) can degrade epitope, protein and particle structures of several viruses including vesicular stomatitis virus (VSV) and Venezuelan equine encephalitis virus [87], [88]. However, since both of the shark sdAb were actually raised by immunization and selection on gamma irradiated Zaire virus it would be logical to reason that BPL might be responsible for epitope destruction in these two cases but not necessarily for llama anti-Zaire sdAb E. Furthermore, gamma irradiated virus has been used to select anti-NP and anti-GP human Fab clones (including the neutralizing KZ52 clone) from Zaire virus infected survivors [89], suggesting retention of critical epitopes is possible by this inactivation method. While BPL inactivated Filovirus material has been shown to be reactive with polyclonal human convalescent serum [90], BPL has also been shown to react with several amino acid residues to form covalently modified BPL adducts [91]. BPL virus inactivation has also been shown to reduce both VSV vaccine immunogenicity [92], the reactivity of 7 out of 23 mouse monoclonal antibodies to Rift Valley Fever virus [93] and to cause a tenfold drop or more in hemagglutination titer and neuraminidase activity of influenza A [94]. In summary, epitope conservation after virus inactivation must be established on a case by case basis, and alternative neutralization methods shown to be less aggressive towards antigenicity of Filoviruses [95] and other viruses [88], [96] should be explored as a means to provide standards suitable for assay path-finding at BSL-2. Whichever inactivation methods are settled upon, carefully demonstrating that a panel of monoclonal affinity reagents has equivalent reactivity to inactivated virus *versus* pretreated live virus will be crucial to show that the authentic antigenic landscapes of these valuable surrogates are conserved.

**Figure 8:**
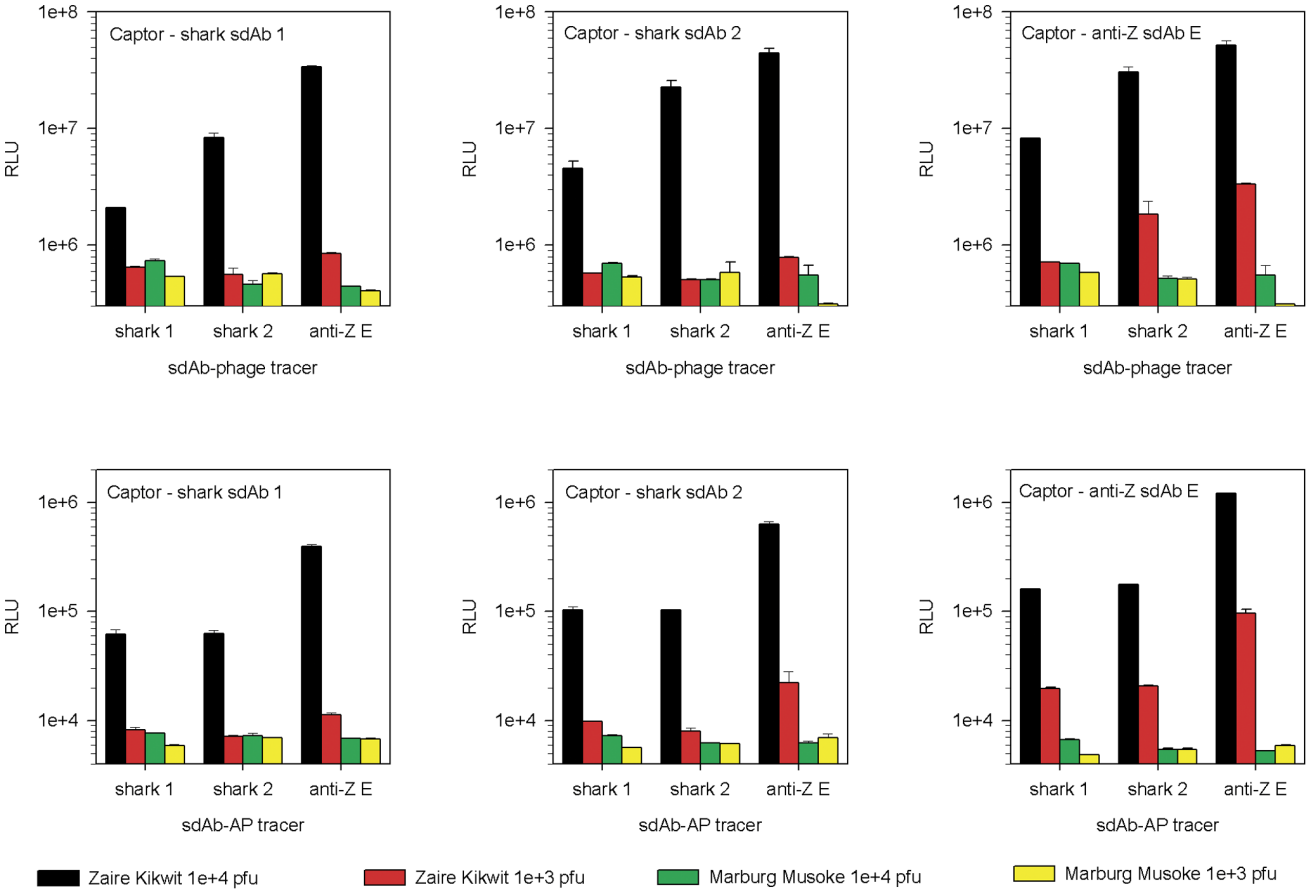
Comparing immune-shark and non-immune llama sdAb reactivities with Zaire virus to determine their relative performance. The two shark sdAb and the llama anti-Zaire sdAb E were assayed side-by-side as captors (indicated by the notation within each bar chart) and as either sdAb-AP fusions or phage displayed sdAb as tracers (indicated by the x axis legend). Two concentrations of target virus Zaire (1e+4 pfu/well, black; 1e+3 pfu/well, red) and control virus Marburg (1e+4 pfu/well, green; 1e+3 pfu/well, yellow) were used as antigen. The error bars represent the maximum and minimum values of duplicate ELISA wells.

We then analyzed the relative abilities of the shark and llama clones to detect live Zaire virus preparations in monoclonal and biclonal affinity reagent sandwich assays where we mixed and matched tracers with captors to approach the issue from 6 different angles. We reveal that the cross-reactive llama anti-Zaire sdAb E excelled whether it was used as captor or tracer with either itself or the shark clones (Fig. 8). Since the shark clones were not as soluble as the llama sdAb we normalized phage titers, using 0.063 µL of phage displayed anti-Zaire sdAb E as opposed to our standard 1 µL used in Figs. 2 and 3, explaining the drop in sensitivity here. That a mediocre semi-synthetic llama sdAb library can rapidly yield a superior binder over using a complex immune route shows that the single-pot llama approach still has much to offer emerging viral threat detection.

### Conservation of the NP C-terminal domain as an *Ebolavirus* Achilles' heel

**Figure 9:**
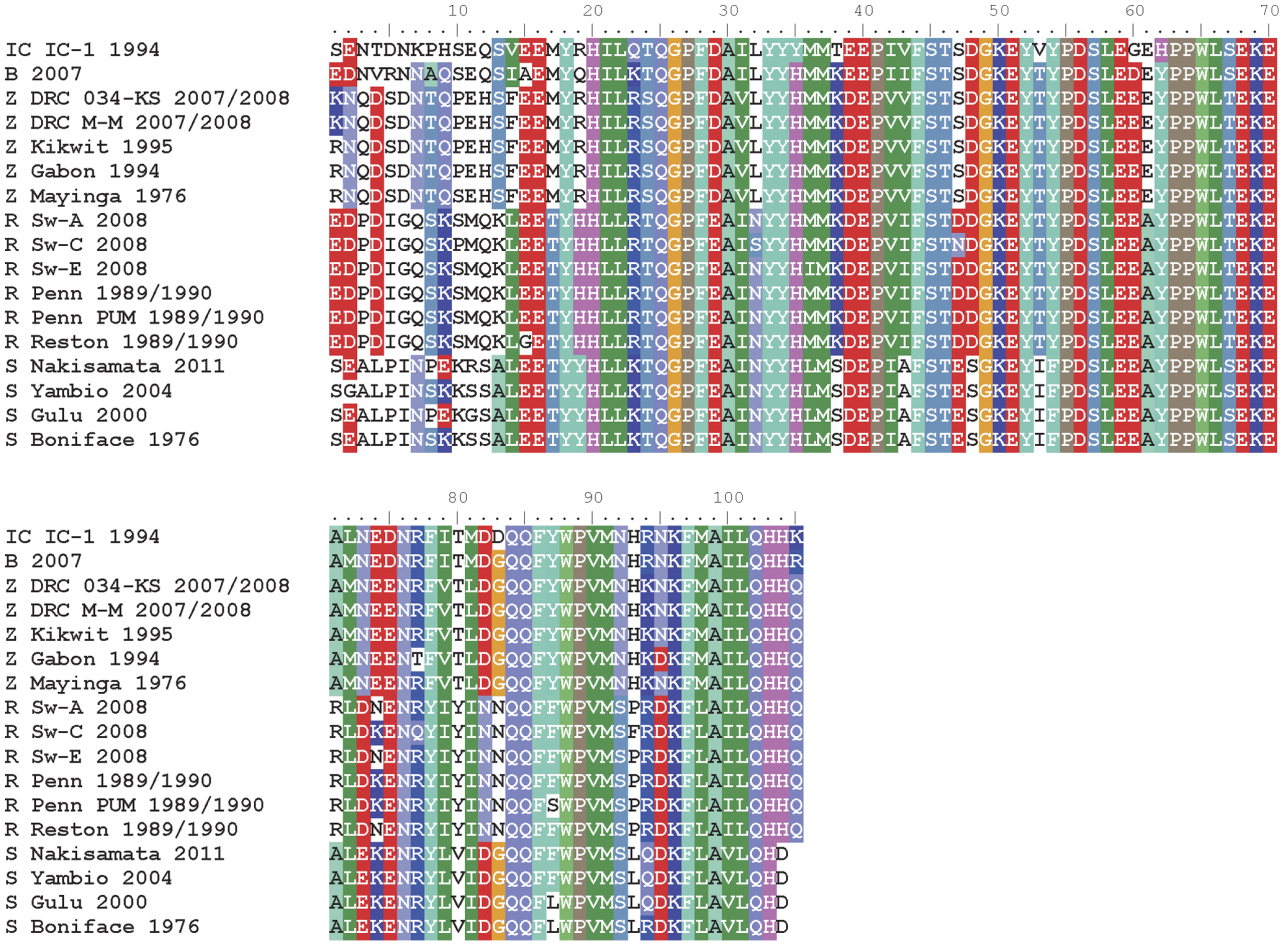
Predicted amino acid sequences of *Ebolavirus* NP C-terminal sequences show high conservation. The last 90 or so amino acids predicted from the *Ebolavirus* NP genes sequenced and deposited in public databases to date are fairly well conserved with occasional polymorphisms at isolated single positions. However, the 15–20 residues before this island of conservation are very different between species, and even show polymorphisms between strains of the same species especially for Sudan virus. Key with Genbank accession number (#), IC = Ivory Coast, B = Bundibugyo, Z = Zaire, R = Reston, S = Sudan: IC IC-1 1994, #NC_014372.1; B 2007, #NC_014373.1; Z DRC 034-KS 2007/2008, # HQ613402.1; Z DRC M-M 2007/2008, # HQ613403.1; Z Kikwit 1995, # AF054908.1; Z Gabon 1994, # Y09358.1; Z Mayinga 1976, # JQ352763.1; R Sw-A 2008, #FJ621583.1; Sw-C 2008, #FJ621584.1; Sw-E 2008, #FJ621585.1; R Penn 1989/1990, #AF522874.1 (USAMRIID); R Penn PUM (Philipps-University Marburg) 1989/1990, #AY769362; R Reston 1989/1990; S Nakisamata 2011, #JN638998.1; S Yambio 2004, #EU338380.1; S Gulu 2000, # Y729654.1; S Boniface 1976, #AF173836.1.

An alignment of the predicted C-terminal domains of *Ebolavirus* NP sequences deduced from nucleotide sequences currently available from Genbank indicate a high degree of conservation, especially in the last 85 or so amino acids (Fig. 9) suggesting critical function(s) in the replication cycle. The last 50 amino acids of NP have been shown to be vital for incorporation into VLP [97] and are thought to involve contacts with the major matrix protein VP40 [61] which drives particle formation [76]. Since NP is an internal antigen it is even less likely to be under antibody based selective pressure to evolve to escape neutralizing antibodies that might pose a problem for tests based upon neutralizing GP antibodies. Though *Ebolavirus* has been shown to adapt and “fine-tune” itself to replicate more effectively in cell culture or laboratory animals [98], [99], [100], [101], mutations are primarily in VP24 and GP with a single coding change detected at Phe>Leu (amino acid #14 in our figure) in guinea-pig adapted virus NP [100] at the border of variable and conserved C-terminal region. There is also a variant at aa#87 deep in the conserved region between two Reston viruses (strain Pennsylvania) grown at two different laboratories, again highlighting local variations in cultivation conditions and passaging can potentially impact the virus. The propensity of *Ebolavirus* to evolve during natural outbreaks has been limited [102] with “remarkable sequence conservation” [63] between different time points in an outbreak [103], [104], between distinct outbreaks of the same species [105], [106], [107], and different clinical outcomes [103], [104], [106]. It will be interesting to see if conservation is retained with increased sampling of animals during human outbreaks [108], animal die offs [109] and novel transmission routes [110] combined with the discovery of new hosts and advances in viral genome sequencing to comprehensively cover the NP gene of all *Ebolavirus* strains.

### Conclusions

A potent molecular magnet for single domain antibodies, polyvalent display, ease of predictive recombinant expression, and selective pressure to remain conserved make the C-terminal domain of *Ebolavirus* a serendipitous and ideal affinity handle for enabling monoclonal affinity reagent sandwich assays (MARSA). Though our focus here is to path find novel routes to better enable rapid detection and diagnostic avenues we speculate on two other potential opportunities given the advantages of recombinant sdAb over conventional IgG. Firstly, since the NP C-terminal domain also interacts with at least one other viral protein and potentially host protein(s) for assembly and budding, we propose these sdAb may also make ideal intrabody candidates to explore disruption of critical interactions during the viral replication cycle [111]. Secondly, the ability of antigen bound sdAb to chaperone hard to crystallize proteins to form high quality crystals for X-ray diffraction [47], [112] may help the atomic structure of polymeric *Ebolavirus* NP to be solved.

## Materials and Methods

### General Methods and Reagents

Recombinant DNA methods were according to established procedures and employed commercially available reagents; HotStart YieldAce, (Stratagene, La Jolla, CA) was used for PCR amplification unless otherwise noted; restriction enzymes and β-agarase (New England BioLabs, Beverly, MA); T4 DNA ligase, CIP and T4 PNK (Roche, Nutley, NJ), AgarACE (Promega, Madison, WI); GTG low melting temperature agarose, (Lonza, Walkersville, MD); oligonucleotides (Integrated DNA Technologies, Coralville, IA); synthetic NP genes (Genscript, Piscataway, NJ). Assemblies involving PCR amplification or oligonucleotide bridging were sequenced through the inserts and junctions to verify the desired construct. Cloning was typically in XL1-Blue unless otherwise stated.

### Biohazards and Safety

Live Filovirus work was performed within the full-suit BSL-4 laboratory at Texas Biomedical Research Institute, following all local and federal guidelines as part of the Select Agent Program.

### Mammalian Cell lines

Vero E6 (#CRL-1586) and HEK 293T (#CRL-11268) cells were obtained from ATCC.

### Virus Amplification and Purification

*Ebolavirus* Zaire strain Kikwit, Sudan strain Boniface, Ivory Coast strain IC1 and Reston strain Reston were each amplified in 16×225 cm^2^ flasks of Vero cells in Dulbecco's modified essential medium (DMEM) with 5% fetal bovine serum (FBS) plus penicillin and streptomycin for 4 days. The 40 mL supernatants were collected, gently clarified by centrifugation (Allegra 6R, swing-out, 2.5 krpm, 5 min, 4°C) and stored at −80°C. Fresh media was added to the flasks and the amplification left for 4 more days. Day 4 supernatants were thawed at room temperature overnight, day 8 supernatants were gently clarified by centrifugation and all virus was precipitated by addition of 1/5^th^ volume of polyethylene glycol 8000, 2.5 M NaCl at 4°C overnight. Precipitated virus was pelleted (Allegra 6R, 3.5 krpm, 15 min, 4°C), resuspended in a total of 4 mL phosphate buffered saline (PBS) and four 1 mL aliquots centrifuged through 10% (w/v) sucrose, PBS onto 65% sucrose cushions (Beckman L70M, SW40, 20 krpm, 1 h, 4°C). Virus was harvested, diluted to 6 mL and 1 mL aliquots loaded on top of six 20 to 65% continuous gradients, and centrifuged at 20 krpm for 24 h at 4°C. Bands were made visible with a torch, harvested, pooled and dialyzed three times against 300 volumes of PBS before aliquoting and storage at −80°C. For panning we used purified viruses, for sandwich ELISAs and western blots we used 8 day old infected Vero cell culture supernatants clarified by gentle centrifugation, aliquoted and stored at −80°C. Also for western blots we used freeze thaw lysates made by scraping the monolayer into the supernatant, freeze-thawing three times (−80°C to 37°C), gently clarifying the suspension (1 krpm, 10 min, 4°C), aliquoting the supernatant and storing at −80°C. Virus was titrated by plaque formation on duplicate 6 well plates of Vero cells with a 1 h infection, gentle wash and overlay with 2 mL of EMEM with 5% FBS plus penicillin and streptomycin containing 0.6% low melting point Seaplaque GTG agarose. On day 10, 2 mL of 4% formaldehyde was added to each well. The plates were incubated in a closed box at 37°C overnight. The agarose plugs were removed and the monolayer was stained with crystal violet for plaque visualization and counting.

### Antibody Selections and Antibody Production

*In vitro* selection of antibodies was performed following standard panning procedures [42] with minor modifications to ease the process at BSL-4. Five wells of an 8 well strip were each coated with 10 µL of purified virus in 100 µL PBS overnight at 4°C, equivalent to approx. 4e+4 pfu of Zaire, 1.2e+4 pfu of Sudan, 9e+3 pfu Reston and 8e+3 pfu of Ivory Coast. Wells were washed three times with 175 µL PBS and blocked for 1 h with 350 µL 2% Carnation non-fat dried milk (MPBS). Phagemids representing 1000 clones of the Nomad semi-synthetic library [48] were applied to each virus coat in five aliquots of 100 µL MBPS for 30 min with shaking. Wells were then washed with 175 µL of PBS 0.1% Tween-20 (PBST) followed by PBS; washings were 10, 20, 20, 30 each for rounds 1 through 4 respectively. Phagemids were eluted with five aliquots of 100 µL 100 mM triethylamine for 10 min followed by pooling and neutralization with 250 µL 1 M Tris-HCl pH 7.5. 375 µL of the neutralized eluate was added to 10 mL of mid exponential phase XL1-Blue and incubated at 37°C for 30 min before titrating an aliquot while the rest was gently pelleted and plated on 15 cm diameter dishes of solid media. [Mid-exponential phase cells were grown from cryopreserved aliquots at BSL-2 for 1 h at 37°C to enable standardization of each batch and synchronization with events in BSL-4]. Overnight growth on plates at 37°C was followed by scraping large plates for glycerol stocking and liquid culture (2xYT plus 2% glucose) in plastic baffled flasks at 37°C for M13K07 superinfection and display with induction by addition of IPTG to 1 mM and overnight growth at 30°C. Aliquots of the cultures were clarified by microfuge and kept at −80°C for polyclonal ELISA while 400 µL was mixed with 100 µL of 10% MPBS and used for the next panning round. Polyclonal ELISA was used to monitor antigen specific enrichment of clones and monoclonal ELISA was used to identify positive phage from 24 clones from each round. 24 positive clones were picked from rounds 3 and 4, miniprepped and DNA combined with phenol chloroform before removal from the BSL-4 laboratory *via* the chemical dunk tank for precipitation and sequencing.

Unique clones were subcloned to pecan45 or pecan16 via *Sfi*I/*Sfi*I for soluble expression and purification of sdAb and sdAb-alkaline phosphatase (AP) proteins respectively as described previously [49], [113]. The anti-Sudan sdAb A was modified by splice overlap extension PCR using primers SudRepTop: 5′-ggttcagtgcaggaagggg-3″ and SudRepBot: 5′-ccccttcctgcactgaacc-3′ to mutate the internal amber codon, creating anti-Sudan sdAb B. Monoclonal phage supernatants from the extracted pecan21 phage display clones were generated at 40 mL scale at BSL-2 by retransforming XL1-Blue and superinfecting with M13K07 with IPTG induction (1 mM). Cultures grown at 30°C were clarified by centrifugation, PEG precipitated and phage resuspended in 1 mL of PBS, mixed with 1 mL of glycerol and stored at −80°C until required.

### Viral Capture Assays

100 nM pure sdAb proteins were passively immobilized on ELISA plates (Costar) overnight at 4°C. Wells were washed three times with PBS, blocked with MPBS for 1 h and then transferred to the BSL-4. Virus preparations were made to 0.1% Triton X-100 in 2% MPBS and incubated for 10 min–cross reactivity trials employed 1e+4 pfu per 100 µL well of virus and titrations employed 1e+4 to 1e-3 pfu per 100 µL well. The MPBS was aspirated, virus was applied and the plate shaken for 10 min (Barnstead International model 4625, setting #2). The wells were washed three times with PBST and twice with PBS and then 1 µL of purified phage displayed sdAb was applied in 100 µL MPBS for 10 min. For comparisons of shark and llama antibodies we were careful to normalize the phage according to titer since the shark clones consistently yielded over a log less phage. Wells were washed again and 1/2000 dilution of anti-M13-HRP (GE Healthcare) was applied in MPBS for 10 min, shaken, washed and plates developed with Super Signal ELISA Pico chemiluminescent substrate and read on a Turner Systems Veritas luminometer. For the sdAb-AP cross-reactivity assay, Tris buffered saline (TBS) was used in place of PBS for the incubations and washes, 100 nM sdAb-AP was used as tracer, and development employed Lumiphos substrate with a 2 second integration time.

### PCR Detection of Virus

Virus dilutions starting ten-fold higher than the aliquots employed in the immunoassay titration were made in 250 µL of media (DMEM plus 5% FCS), extracted with 750 µL of Trizol LS, RNA resuspended in 10 µL and stored at −80°C until required. 1 µL corresponding to the actual pfu per well used in the ELISAs was added to a 24 µL reaction of Qiagen OneStep RT-PCR kit kit containing the pan reactive Ebola specific primers (for Ivory Coast, Reston, Sudan and Zaire samples) or Marburg specific primers (for Marburg virus) and subjected to 50 cycles as detailed by Ogawa and colleagues [57]. We used tenfold higher primer concentrations than recommended since that gave us a larger dynamic range of band intensities after 50 cycles over the standard amount. Negative control reactions were no virus (processing media only) and no template (nothing added to RT-PCR reaction). 5 µL was analyzed on a 1% agarose gel alongside 1 kb DNA ladder (Invitrogen, Grand Island, NY).

### Western Blotting of Viral Proteins

500 µL of freeze thaw lysate, representing 4e+5 pfu of Reston, 1e+5 pfu of Ivory Coast, 6e+5 pfu of Sudan and 9e+5 of Zaire viruses and 1 mL of *Marburgvirus* Musoke representing 3e+6 pfu were each combined with an equal volume of Laemmli sample buffer and the mix heated at 100°C for 10 min. After cooling the samples were removed from the BSL-4 *via* the chemical dunk tank, reheated for 1 min and 50 µL electrophoresed on a 12% SDS-PAGE Laemmli gel in pairs of experimental cognate virus and *Marburgvirus* as a negative control. Marker proteins (Biorad, Kaleidoscope Precision Plus Protein Standards) were used to gauge approx. molecular weights. Gels were semi-dry blotted to Immobilon P cut into strips and probed with 100 nM sdAb-AP conjugates in MTBS for 1 h, washed three times with TBST, twice with TBS, developed with Lumiphos and images caught on Fuji Super-RX X-ray film.

For comparing CRP materials with supernatants and freeze thaw lysates, samples were combined with Laemmli sample buffer such that 1.25 µL of CRP materials were loaded on a 10% gel for silver staining and 2.5 µL of Zaire CRP material was loaded on a 10% gel for western blotting with 1/1000 rabbit polyclonal anti-Zaire NP (#0301-012, Integrated BioTherapeutics Inc., Gaithersburg, MD) for 1 h followed by 1/5000 anti-rabbit HRP conjugate (Sigma) with development using SuperSignal West Pico chemiluminescent substrate (Thermo Scientific). Western blotting was also performed using 100 nM sdAb-AP conjugates of anti-Zaire sdAb E, shark 1 sdAb and shark 2 sdAb as probes using Lumiphos development. Our supernatants and freeze-thaw lysates were normalized to CRP pfu loadings for western blots, and normalized to equivalent volumes for silver staining.

### Recombinant Prokaryotic Nucleoproteins

Zaire Kikwit and Marburg Musoke genes were cloned with C-terminal his tags into a tac promoter cytosolic expression vector pecan 42 as described previously [49]. To clone the Sudan Boniface and Reston Reston genes, monolayers of 8 dpi 80 cm^2^ flasks of infected Vero cells were gently washed with serum free medium and then harvested in 8 mL of Trizol for removal from the BSL-4 laboratory. Following extraction, 2 µg of RNA was used in an oligo dT primed RT-PCR reaction to generate cDNA using an Ambion RNA RETROscript kit. 2 µL of the 20 µL reactions were used in Pfu polymerase PCR reactions using either EboSudBonNPfor 5′-aaaacatatggataaacgcgtgagag-3′ and EboSudBonNPback 5′-gccgcgaattcgtcatgttgaagaacggc-3′ for Sudan NP or EboResResNPfor (5′-aaaacatatggatcgtgggaccag-3′) and EboResResNPHisback (5′-gccgcgaattcctgatggtgctgcagatt-3′) for the Reston NP. The products were digested with *Nde*I (partial), *Eco*RI and cloned into similarly digested pecan42, clones picked and mapped. Six correct clones of each were sequenced to identify a consensus matching the Genbank sequences with forward and reverse vector sequencing primers AHX76 and AHX89 in concert with gene specific primers for the Sudan gene; EboSudBon;NP500 (5′gcgtgcctggaaaaagtac-3′), NP1000 (5′-ggtgtgaatgtaggggagc-3′), NP1500 (5′-caccagacagggggcagaac-3′);and for the Reston gene EboResRes;NP500 (5′-cttgcttggaaaaagtccagc-3′), NP1000 (5′-gttaatgttggtgagcagtatc-3′) and NP1500 (5′-caggacagctcaccacaatc-3′). Genes encoding the Ivory Coast IC-1 and Bundibugyo NP were ordered from Genscript based on Genbank sequences and have *Nde*I based initiation codons and C-terminal EcoRI sites for cloning as above. After sub-cloning, these genes were also sequenced to confirm they were as expected with AHX76, AHX89 and either ICNP500for (5′-ctagttgtcggagaaaaagcc-3′), ICNP1000for (5′-cacacggcagtaccctggc-3′), ICNP1500for (5′-gatcaccgaccgtcaagttc-3′), ICNP2000for (5′caaggaccatttgatgccatcc-3′) or BunNP500for (5′-ggttgtcggagaaaaggcc-3′), BunNP1000for (5′-agcactctggctggagtc-3′), BunNP1500for (5′-cccggctcaaaacacgcc-3′), BunNP2000for (5′-cacaaggaccttttgatgcc-3′).

*E. coli* Tuner pRARE bearing each of the NP gene expression vectors was grown to 20 mL scale in glucose free terrific broth and induced with 1 mM IPTG for 3 h at 25°C. 20 ODcm^−1^ at A600 nm of each culture was pelleted and resuspended in 2 mL of PBS with Complete protease inhibitor cocktail, subjected to mini-beadbeating at 4°C for 4 pulses of 3 min with 1 min intervals and stored at −80°C until required. 50 µL was combined with an equal volume of Laemmli sample buffer, boiled for 3 min, and 10 µl electrophoresed on a 10% SDS- PAGE gel alongside Kaleidoscope markers and semi-dry blotted to Immobilon P or Coomassie stained. For the westerns 1/1000 anti-His-1 HRP conjugate (Sigma) was initially used to probe the blots to ensure all proteins were expressed at the expected molecular weights with the blot developed with SuperSignal West Pico Chemiluminescent substrate. sdAb AP probing for the recombinant NP was as for virus antigen probing as described above.

Window deletions of the Zaire NP gene were made *in situ* using the Stratagene Quick-Change Multi Site-Directed Mutagenesis kit and oligonucleotides designed to loop out approx. 315 nucleotides to remove 105 predicted amino acids. Oligonucleotides were EBOZNPdel1 (5′-tctagagaaggagatatacatatggaagtcaagaagcgtgatggagtg-3′), EBOZNPdel2 (5′-ttggaagggcacgggttccgttttctcctaatacaccaagggatgcac-3′), EBOZNPdel3 (5′-cgaacaaattttttgatcaaatttattgcactcggagtcgccacagca-3′), EBOZNPdel4 (5′-ggtcttttccctcaactatcagcagacgacattccctttccaggaccc-3′)

EBOZNPdel5 (5′-acaagtggacattacgatgatgatgtcccaggccctcacagaacaatc-3′),

EBOZNPdel6 (5′-acacaatccaggccaactcaaaataggaaccaggacagtgacaacacc-3′),

EBOZNPdel7 (5′-gatcaggaccacactcaagaggccgaattcgcggccgcagtcgaccat-3′).

Clones revealing deletion by agarose gel analysis were sequenced to ensure no other changes occurred in the expression cassette with AHX76, AHX89 and the relevant Zaire NP sequencing primers. Deletion 1 and 2 used the internal primer EBOZNPdel5for (5′-gtcccaggccctcacagaac-3′), deletions 3, 6 and 7 used EBOZNPdel4for (5′-gacgacattccctttcca-3′) and deletions 4 and 5 used EBOZNPdel2for (5′-ctcctaatacaccaagggat-3′). Lysates of crude *E. coli* were made and processed as described for the full-length NP genes.

### Recombinant Eukaryotic Nucleoproteins

pcDNA3.1- (Invitrogen) was modified to enable directional cloning of genes using *Sfi*I by ligating an oligonucleotide bridge of phosphorylated and annealed primers cDNASfiTOP (5′-ctagcggtaccggcccagccggcctaaaaaaaggcctcgggggccgcggccgca-3′) and cDNASfiBOTTOM (5′-agcttgcggccgcggcccccgaggcctttttttaggccggctgggccggtaccg-3′) between the *Nhe*I and *Hin*dIII sites. The resulting vector pcDNASfi was sequenced with primers cDNAfor (5′-cgaaattaatacgactcactatggg-3′) and cDNAback (5′-caactagaaggcacagtcgaggc-3′). Human codon optimized NP genes with 5′-Sfi flank prior to the start codon designed to form a Kozak consensus (5- ggcccagccggccgccacc…) and 3′-Sfi flank (…ggcctcgggggcc- 3′) were ordered for

*Ebolavirus* Zaire strain Kikwit, Sudan strain Boniface, Reston strain Reston, Ivory Coast strain IC1 and *Marburgvirus* Musoke from Genscript. After subcloning into pcDNASfi and *Sfi*I restriction mapping, two clones of each gene were sequenced with cDNAfor, cDNAback, HuOptEBONP′s655 (5′-ctgctgattcaccaggg-3) for the *Ebolavirus* genes and HuOptMBGNP650 (5′-gattccatcattagcaattc-3′) for the *Marburgvirus* NP gene. Following checkerboard optimization of the method in small-scale [114], 10 µg of Qiagen miniprep DNA in 100 µL water was combined with 1150 µL DMEM, while 40 µL of 1 mg/mL branched PEI was combined with 1210 µL DMEM before mixing the two and allowing to sit at room temperature for 20 min in a 35 mm tissue culture dish. The DNA-PEI complex was gently added to 80% confluent HEK 293T cells grown in DMEM plus 5% FBS that were seeded 18 h previously on a 10 cm dish and left on cells [115]. Transfection efficiencies were monitored with a parallel plate with pCMVBgal (BD) followed by staining with X-gal to reveal typically 50% blue. On day 3 post transfection, cells were washed gently with TBS, then collected in 4 mL of ice-cold 10 mM Tris-Cl pH 7.5, 150 mM NaCl, 1 mM EDTA, 1% NP40 plus Roche Complete protease inhibitor cocktail. The plates were left at 4°C for 30 minutes. The cells were collected by pipetting up and down with a P1000 pipette and then aliquoted into two 2 mL tubes per plate and centrifuged at 20,000 g for 10 minutes. The supernatant was decanted, like tubes combined, and 600 µL of glycerol was added and mixed well before dispensing into usable aliquots. Storage was at −80°C. To assay for expression 10 µL of lysate was combined with an equal volume of Laemmli sample buffer, electophoresed and processed as for the recombinant *E. coli* NP genes. 5 µL was used directly in sandwich assays performed as for the virus capture assays but without additional detergent incubation required.

### Shark sdAb

Synthetic genes encoding the two shark sdAb isolated from an immune library by Goodchild and colleagues [56] were obtained from Genscript from framework 1 to framework 4 (RVD…TVN). Sequences were *E. coli* expression optimized based on the published amino acid sequence and had flanks to enable cloning into our phage display, soluble sdAb and sdAb-AP expression vectors. Proteins were purified and quantified as described previously while phage was grown and precipitated as previously described. The phage was titrated and used in the sandwich ELISAs at an equivalent 2e8 cfu/well as tracer.

### Inactivated Virus Antigens

Viral supernatants that were collected by centrifugation, titrated and then inactivated with beta-propiolactone and gamma irradiation were kindly provided by the Critical Reagents Program of the Department of Defense. Titers provided were (in pfu/mL); Reston, 1.1e+7; Ivory Coast, 4.1e+5; Zaire, 1.7e+7; Marburg, 3.6e+6; neither Sudan nor Bundibugyo were available. Materials were thawed once, aliquoted to smaller volumes and stored at −80°C until used. 90 µL of each reagent was mixed with 810 µL of 0.1% Triton in MPBS before application of duplicate 100 µL volumes to the sandwich capture plate, with a tenfold dilution, followed by processing as per our standard virus capture ELISA described above.

## Supporting Information

Figure S1 ***Ebolavirus*** **antigen capture assays employing alkaline phosphatase sdAb fusion proteins as tracers.** SdAb specific for A) Ivory Coast, B) Reston, C) Sudan and D) Zaire were passively immobilized to ELISA wells as captors for 1e+4 pfu of Ivory Coast (black), Reston (red) Sudan (green), Zaire (yellow) or Marburg (blue) viruses. Detection used the same sdAb clone as a fusion to hyperactive *E. coli* alkaline phosphatase [49], [118] followed by chemiluminescent substrate. The error bars represent the maximum and minimum values of duplicate ELISA wells.
(PDF)

Figure S2 **Recognition of western blotted recombinant NP confirms antigen identity and predicts reactivity towards** ***Ebolavirus*** **Bundibugyo.** Each sdAb-AP fusion was used to probe total *E. coli* lysates expressing C-terminally His-tagged NP of Bundibugyo (B), Ivory Coast (IC), Reston (R), Sudan (S), Zaire (R), and negative control Marburg (M). A western blot probed with anti-His-HRP (top left) served to confirm all proteins were expressed while a Coomassie stained gel (bottom right) indicated the total amount of lysates loaded were equivalent, though Reston NP was relatively poorly expressed (molecular weight markers-mk).
(PDF)

Figure S3 **HEK 293T recombinant NP lysates as predictors of future strain reactivity in MARSAs.** A) Coomassie stained SDS-PAGE analysis of human optimized NP genes expressed in HEK 293T cells showing modest 100 kDa bands with lower expression in Reston samples [Bundibugyo (B), Ivory Coast (IC), Reston (R), Sudan (S), Zaire (R), and negative control Marburg (M), molecular weight markers (mk). B) Western blot of lysates probed with cross-reactive anti-Zaire sdAb E as a sdAb-AP fusion confirms expression of NP. The panels of sdAb specific for C) Ivory Coast, D) Reston, E) Sudan and F) Zaire viruses were used as captors while matching phage displayed sdAb were used as tracers. The error bars represent the maximum and minimum values of duplicate ELISA wells.
(PDF)

Table S1 Frequency of occurrence of sdAb clones from 24 monoclonal positives picked from rounds 3 and 4. Sudan clone A refers to the parental clone while Sudan B refers to the repaired clone. Zaire clone B was not characterized due to oversight.
(PDF)
