# Supplementary figures and images for: Ebolavirus Nucleoprotein C-Termini Potently Attract Single Domain Antibodies Enabling Monoclonal Affinity Reagent Sandwich Assay (MARSA) Formulation
Source: PLoS One. 2013 Apr 5;8(4):e61232. doi: 10.1371/journal.pone.0061232 (PMC3618483; doi:10.1371/journal.pone.0061232)

Figure S1

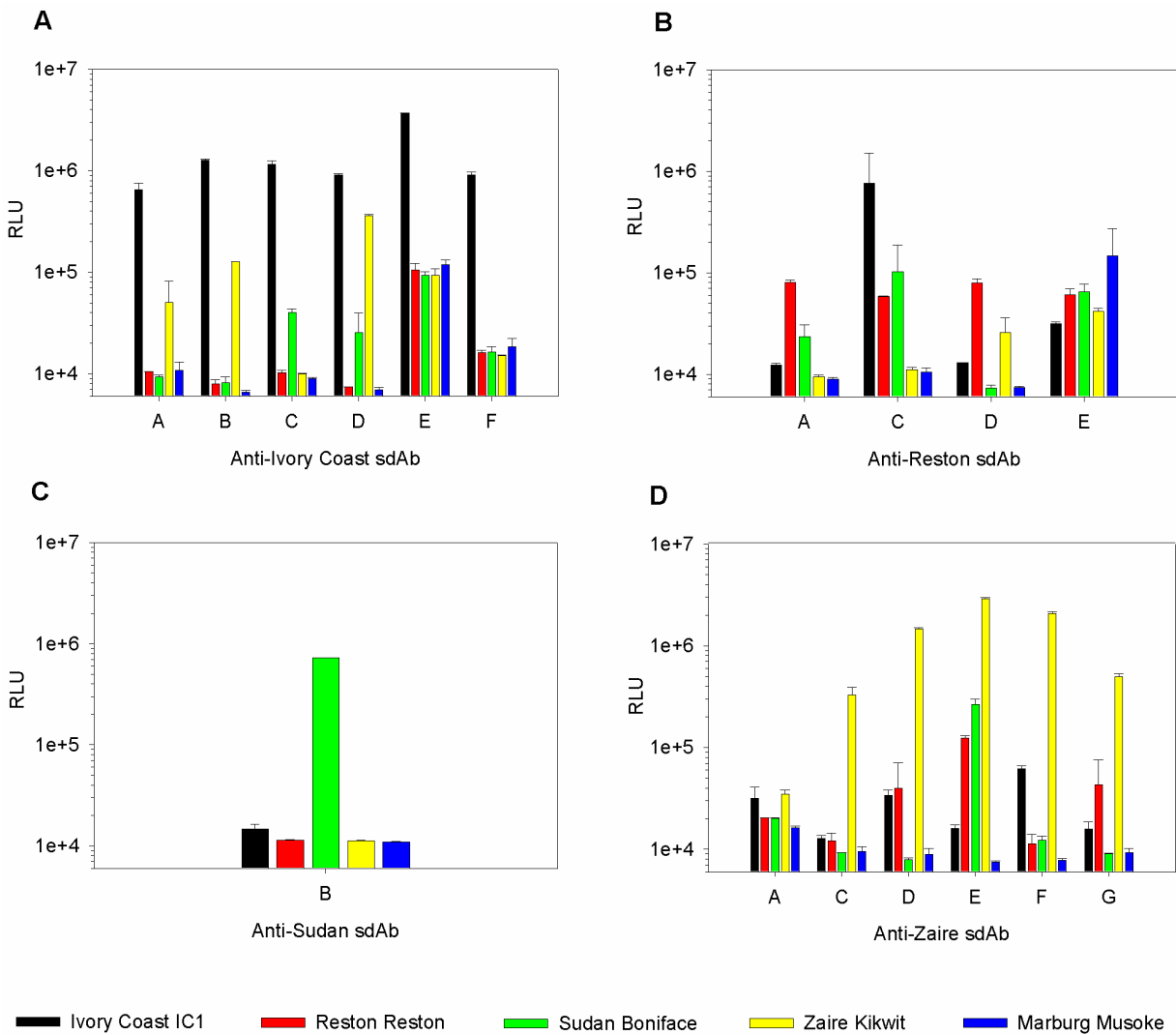

Supplement: Figure S1 — Ebolavirus antigen capture assays employing alkaline phosphatase sdAb fusion proteins as tracers. SdAb specific for A) Ivory Coast, B) Reston, C) Sudan and D) Zaire were passively immobilized to ELISA wells as captors for 1e+4 pfu of Ivory Coast (black), Reston (red) Sudan (green), Zaire (yellow) or Marburg (blue) viruses. Detection used the same sdAb clone as a fusion to hyperactive E. coli alkaline phosphatase [49], [118] followed by chemiluminescent substrate. The error bars represent the maximum and minimum values of duplicate ELISA wells. (PDF) [file pone.0061232.s001.pdf]

**Figure S2**

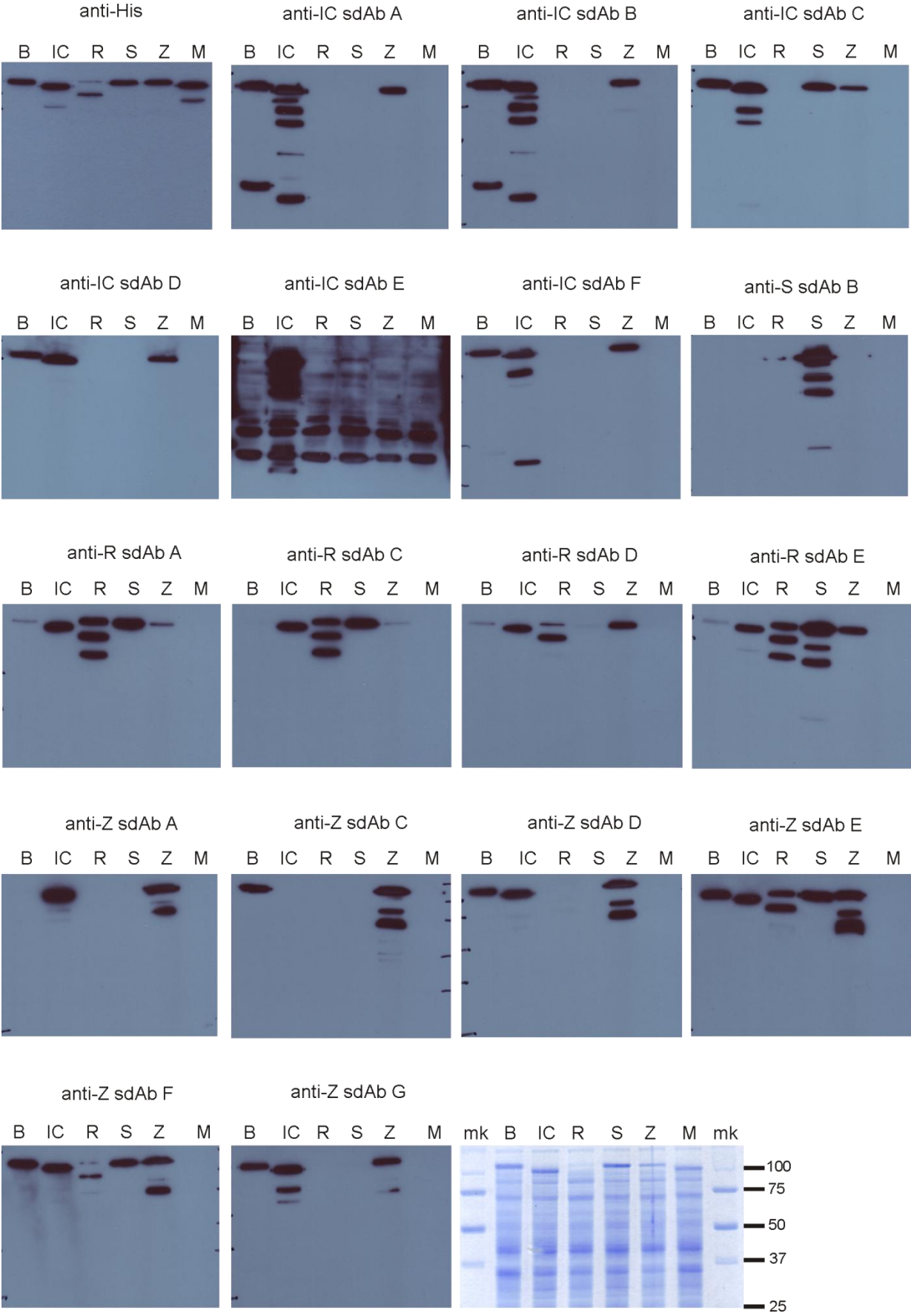

Supplement: Figure S2 — Recognition of western blotted recombinant NP confirms antigen identity and predicts reactivity towards Ebolavirus Bundibugyo. Each sdAb-AP fusion was used to probe total E. coli lysates expressing C-terminally His-tagged NP of Bundibugyo (B), Ivory Coast (IC), Reston (R), Sudan (S), Zaire (R), and negative control Marburg (M). A western blot probed with anti-His-HRP (top left) served to confirm all proteins were expressed while a Coomassie stained gel (bottom right) indicated the total amount of lysates loaded were equivalent, though Reston NP was relatively poorly expressed (molecular weight markers-mk). (PDF) [file pone.0061232.s002.pdf]

Figure S3

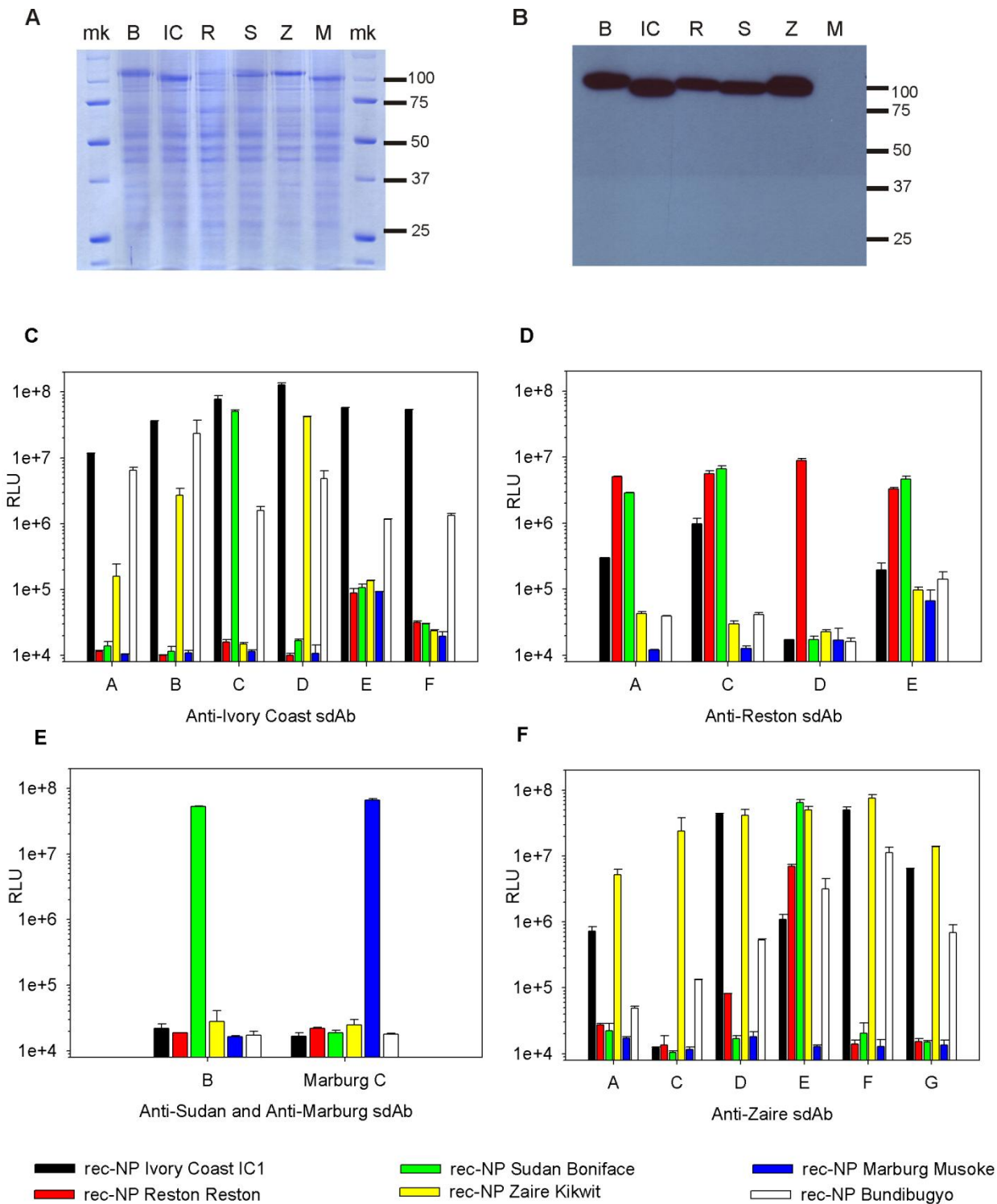

Supplement: Figure S3 — HEK 293T recombinant NP lysates as predictors of future strain reactivity in MARSAs. A) Coomassie stained SDS-PAGE analysis of human optimized NP genes expressed in HEK 293T cells showing modest 100 kDa bands with lower expression in Reston samples [Bundibugyo (B), Ivory Coast (IC), Reston (R), Sudan (S), Zaire (R), and negative control Marburg (M), molecular weight markers (mk). B) Western blot of lysates probed with cross-reactive anti-Zaire sdAb E as a sdAb-AP fusion confirms expression of NP. The panels of sdAb specific for C) Ivory Coast, D) Reston, E) Sudan and F) Zaire viruses were used as captors while matching phage displayed sdAb were used as tracers. The error bars represent the maximum and minimum values of duplicate ELISA wells. (PDF) [file pone.0061232.s003.pdf]
