# Supplementary material for: Ebolavirus Nucleoprotein C-Termini Potently Attract Single Domain Antibodies Enabling Monoclonal Affinity Reagent Sandwich Assay (MARSA) Formulation
Source: PLoS One. 2013 Apr 5;8(4):e61232. doi: 10.1371/journal.pone.0061232 (PMC3618483; doi:10.1371/journal.pone.0061232)
Supplement: Table S1 — Frequency of occurrence of sdAb clones from 24 monoclonal positives picked from rounds 3 and 4. Sudan clone A refers to the parental clone while Sudan B refers to the repaired clone. Zaire clone B was not characterized due to oversight. (PDF) [file pone.0061232.s004.pdf]

Table S1

Ebolavirus Species	sdAb	Frequency
**Reston**	A	3/24
	C	10/24
	D	10/24
	E	1/24
**Sudan**	A (B)	24/24
**Ivory Coast**	A	5/26
	B	11/26
	C	6/26
	D	1/26
	E	1/26
	F	2/26
**Zaire**	A	3/24
	C	11/24
	D	2/24
	E	1/24
	F	3/24
	G	1/24
	(B)	(3/24)
